# Canonical TSH Regulation of Cathepsin-Mediated Thyroglobulin Processing in the Thyroid Gland of Male Mice Requires Taar1 Expression

**DOI:** 10.3389/fphar.2018.00221

**Published:** 2018-03-20

**Authors:** Maria Qatato, Joanna Szumska, Vladislav Skripnik, Eddy Rijntjes, Josef Köhrle, Klaudia Brix

**Affiliations:** ^1^Department of Life Sciences and Chemistry, Jacobs University Bremen, Bremen, Germany; ^2^Institut für Experimentelle Endokrinologie, Charité – Universitätsmedizin Berlin, Freie Universität Berlin–Humboldt-Universität zu Berlin–Berlin Institute of Health, Berlin, Germany

**Keywords:** cathepsins, morphometry, thyroglobulin, thyroid stimulating hormone receptor, trace amine-associated receptor 1

## Abstract

Trace amine-associated receptor 1 (Taar1) has been suggested as putative receptor of thyronamines. These are aminergic messengers with potential metabolic and neurological effects countering their contingent precursors, the thyroid hormones (THs). Recently, we found Taar1 to be localized at the primary cilia of rodent thyroid epithelial cells *in vitro* and *in situ*. Thus, Taar1 is present in a location of thyroid follicles where it might be involved in regulation of cathepsin-mediated proteolytic processing of thyroglobulin, and consequently TH synthesis. In this study, *taar1* knock-out male mice (*taar1*^-/-^) were used to determine whether Taar1 function would entail differential alterations in thyroid states of young and adult animals. Analyses of blood serum revealed unaltered T_4_ and T_3_ concentrations and unaltered T_3_-over-T_4_ ratios upon Taar1 deficiency accompanied, however, by elevated TSH concentrations. Interestingly, TSH receptors, typically localized at the basolateral plasma membrane domain of wild type controls, were located at vesicular membranes in thyrocytes of *taar1*^-/-^ mice. In addition, determination of epithelial extensions in *taar1*^-/-^ thyroids showed prismatic cells, which might indicate activation states higher than in the wild type. While gross degradation of thyroglobulin was comparable to controls, deregulated thyroglobulin turnover in *taar1*^-/-^ mice was indicated by luminal accumulation of covalently cross-linked thyroglobulin storage forms. These findings were in line with decreased proteolytic activities of thyroglobulin-solubilizing and -processing proteases, due to upregulated cystatins acting as their endogenous inhibitors *in situ*. In conclusion, Taar1-deficient mice are hyperthyrotropinemic in the absence of respective signs of primary hypothyroidism such as changes in body weight or TH concentrations in blood serum. Thyrocytes of *taar1*^-/-^ mice are characterized by non-canonical TSH receptor localization in intracellular compartments, which is accompanied by altered thyroglobulin turnover due to a disbalanced proteolytic network. These finding are of significance considering the rising popularity of using TAAR1 agonists or antagonists as neuromodulating pharmacological drugs. Our study highlights the importance of further evaluating potential off-target effects regarding TSH receptor mislocalization and the thyroglobulin processing machinery, which may not only affect the TH-generating thyroid gland, but may emanate to other TH target organs like the CNS dependent on their proper supply.

## Introduction

Trace amine-associated receptor 1 (Taar1) is a G protein coupled receptor (GPCR) that was shown to be involved in regulation of dopaminergic neurotransmission in rodents (Wolinsky et al., 2007; Lindemann et al., 2008; Espinoza et al., 2011; Revel et al., 2011, 2012a; Leo et al., 2014; Harmeier et al., 2015). Subsequently, Taar1 was considered as a drug target for treatment of alcohol and drug addiction, as well as Parkinson’s disease and schizophrenia (Bradaia et al., 2009; Galley et al., 2012; Revel et al., 2012b; Lynch et al., 2013; Alvarsson et al., 2015; reviewed in Grandy, 2007, 2014; Grandy et al., 2016; Berry et al., 2017). In the context of neuro-modulation, *taar1*^-/-^ mouse models were developed to study schizophrenia particularly, but also other neuropsychiatric disorders. The *taar1*^-/-^ mouse model exhibited elevated sensitivity to amphetamines in comparison to wild type (WT) littermates, which manifested in enhanced locomotor activity. This was correlated with higher dopamine and norepinephrine release in the striatum upon amphetamine treatment, as well as a significant increase in striatal expression of high-affinity dopamine receptors, all of which are regarded as characteristic symptoms of schizophrenia (Wolinsky et al., 2007; Lindemann et al., 2008). Otherwise, the *taar1*^-/-^ mouse proved comparable to WT controls in terms of development and growth, as well as tissue morphogenesis, as far as this was investigated.

Hence, Taar1 and the *taar1*^-/-^ mouse models have been studied in relation to behavior and the central nervous system, but were not yet investigated in detail with regard to the thyroid gland and possible regulation of its functions as thyroid hormone (TH)-generating endocrine organ. Because TH play a crucial role in brain development, and were shown to be decreased in various neuropsychiatric disorders (Bauer et al., 2008), including depression, Parkinson’s disease, and schizophrenia, we investigated the thyroid phenotype of *taar1*^-/-^ mice. Since we have previously shown that Taar1 is localized at primary cilia of thyroid epithelial cells *in vitro* and *in situ* (Szumska et al., 2015), the present study asks in particular whether Taar1 plays a role in thyroid morphology and its functional activity.

Taar1 at primary cilia of the apical plasma membrane of thyroid epithelial cells is exposed to the pH neutral and oxidizing milieu of the thyroid follicle lumen, in which thyroglobulin (Tg) is stored. The apical plasma membrane domain of thyrocytes is the site of Tg secretion, upon which it is iodinated to preform TH, and is then stored in the lumen of thyroid follicles in covalently cross-linked form. In addition, partial degradation by Tg-processing cathepsins B, K, and L for solubilization and initial TH liberation, and subsequent endocytosis of Tg happen at the apical plasma membrane domain, too, i.e., in direct vicinity of the Taar1-bearing cilia of thyrocytes (Brix et al., 1996; Tepel et al., 2000; Jordans et al., 2009; reviewed in Dauth et al., 2011). Currently, the site(s) of thyronamine production remain(s) controversial (Glossmann and Lutz, 2017) as both the thyroid gland (Hackenmueller et al., 2012), the gastrointestinal mucosa and other potential tissues (Hoefig et al., 2016) have been proposed to provide these aminergic ligands, which were shown to activate TAAR1 *in vitro* (Scanlan et al., 2004). Thus, it is plausible that the thyroid follicle lumen may be providing ligands that activate Taar1 at cilia of the apical plasma membrane domain of thyrocytes (Szumska et al., 2015), thereby implicating that Taar1 could be involved in the regulation of thyroid gland functions, namely Tg degradation and, consequently, TH liberation. Therefore, we were interested in testing whether Taar1 is required not only for thyroid tissue morphogenesis, but also for regulation of Tg processing and the resulting serum TH status, which are important aspects of thyroid gland function in maintaining proper TH supply to peripheral and central target organs, including the CNS.

Classical regulation of the thyroid gland involves the hypothalamic–pituitary–thyroid (HPT) axis, whereby low TH concentrations trigger a negative feedback operating in parallel and resulting in thyroid releasing hormone (TRH) release from the hypothalamus and thyroid stimulating hormone (TSH) release from the pituitary gland (for reviews, see Fekete and Lechan, 2014; Ortiga-Carvalho et al., 2016). Circulating TSH binds to its receptors (human TSHR/mouse Tshr) expressed at the basolateral plasma membrane of thyrocytes. Ligand binding on TSHR induces G_qα_ and G_sα_ signaling pathways. Activation of G_qα_ rapidly culminates in relocation of Tg-processing cathepsins to the apical plasma membrane, where they are released into the thyroid follicle lumen to initiate Tg solubilization and TH liberation (Brix et al., 1996; Linke et al., 2002). This is completed by re-internalization of partially degraded Tg molecules for lysosomal degradation and exhaustive TH liberation (Friedrichs et al., 2003; Jordans et al., 2009). In contrast and subsequently, the long-term effect of TSH stimulation entails enhanced secretion of *de novo* synthesized Tg into the follicular lumen (reviewed in Brix et al., 2001; Dauth et al., 2011). Thus, any disturbances in TSH regulation of thyrocytes will potentially translate into alterations in levels of Tg-processing proteases and, therefore, the degree of Tg processing and degradation, which would eventually affect TH concentrations in the blood serum. Accordingly, this study included investigations on the Tshr to determine the effect of Taar1 deficiency on thyroid function and its regulation in young and older adult male mice.

## Materials and Methods

### Animals, Thyroid Tissue Sampling, and Cryosectioning

In this study, male mice were used to eliminate potential discrepancies due to hormonal fluctuations in females. *Taar1*^-/-^ and C57BL6/J WT mice were kept in the animal facility of Jacobs University Bremen, Germany. The founder *taar1*^-/-^ mice were provided by Dr. David K. Grandy (Oregon Health and Science University, Portland, OR, United States), and genotyped as previously described (Szumska et al., 2015). Mice were housed under standard conditions, with a 12 h/12 h light/dark cycle and *ad libitum* water and food. Testing was conducted in accordance with institutional guidelines in S1-laboratories of Jacobs University Bremen (SfAFGJS Az. 513-30-00/2-15-32 and Az. 0515_2040_15).

Body weight was assessed for *n* = 52 and 16 biological replicas for WT and *taar1*^-/-^ male animals, respectively, from 8 to 44 weeks of age. Young (5–8 months old) and older adult (10–15 months old) male mice were euthanized by CO_2_ inhalation. Blood sampling was in the mornings from 9:30 am to 12:30 pm, i.e., 3:30 to 6:30 h:min after the start of the light cycle. Afterward, perfusion was carried out through the heart with 0.9% NaCl including 0.4 IU heparin per mL. The resected thyroid gland tissue was either snap-frozen in liquid nitrogen and stored at -80°C until used, or fixed in 4% paraformaldehyde (PFA) in 200 mM HEPES, pH 7.4, and left overnight at 4°C. Cryo-preservation was carried out by incubation in Tissue Freezing Medium (Jung, through Leica Microsystems, Nussloch, Germany) overnight at 4°C, then frozen and stored at -20°C until sectioning on a cryostat (Leica CM1900, Leica Microsystems) into 5 μm-thick transverse sections and thaw-mounting on microscope slides.

### Indirect Immunofluorescence

Residual embedding solution was washed out by overnight-incubation in calcium- and magnesium-free PBS (CMF-PBS), composed of 0.15 M NaCl, 2.7 mM KCl, 1.5 mM NaH_2_PO_4_, 8.1 mM Na_2_HPO_4_ at pH 7.4, at 4°C, followed by blocking with 3% bovine serum albumin (BSA; Carl Roth GmbH, Karlsruhe, Germany) in CMF-PBS for 1 h at 37°C. The sections were then incubated with primary antibodies diluted in 0.1% BSA in CMF-PBS, overnight at 4°C (**Table 1**). After washing with 0.1% BSA in CMF-PBS, the sections were incubated with Alexa Fluor^®^ 488- or Alexa Fluor^®^ 546-conjugated secondary antibodies for 1 h at 37°C (1:200; Molecular Probes, Karlsruhe, Germany) together with 5 μM of the nuclear counter-stain Draq5^TM^ (BioStatus Limited, Shepshed, Leicestershire, United Kingdom). Epithelial cells were stained with HCS CellMask^TM^ Orange for 1 h at 37°C (1:1000, Molecular Probes, H32713). Glycosylated tissue components were stained with the biotinylated lectin concanavalin A from *C. ensiformis* (ConA; Sigma-Aldrich, C2272) at 10 μg/mL for 30 min at 4°C, followed by incubation with Alexa Fluor^®^ 546-conjugated streptavidin (Molecular Probes, Karlsruhe, Germany, S-11225) as the secondary ConA detection label. Specific antibodies were omitted in negative controls. After washing with CMF-PBS and deionized water, the sections were mounted with embedding medium consisting of 33% glycerol, and 14% Mowiol in 200 mM Tris-HCl, pH 8.5 (Hoechst AG, Frankfurt, Germany).

**Table 1:** Antibodies used in this study.

Antigen	Specificity	Company/provider	Catalog number	Dilution in immuno-fluorescence	Dilution in immuno-blotting
β-Tubulin	Rabbit anti-human	Abcam	#ab6067	_	1:1000
Cathepsin B	Goat anti-mouse	Neuromics	#GT15047	1:100	1:1000
Cathepsin D	Rabbit anti-human	Calbiochem	#IM-16	1:10	1:250
Cathepsin L	Goat anti-mouse	Neuromics	#GT15049	1:100	1:1000
Collagen IV	Rabbit anti-mouse	Novotech	#CO20451	1:100	_
Cystatin C	Rabbit anti-mouse	Dr. Magnus Abrahamson, Lund, Sweden	_	1:25	_
Cystatin D	Rabbit anti-mouse	Dr. Magnus Abrahamson, Lund, Sweden	_	1:25	_
Monocarboxylate transporter 8 (Mct8, Slc16A2)	Rabbit anti-human	Atlas antibodies	#HPA003353, lot A61491	1:200	_
Thyroglobulin	Rabbit anti-bovine	Brix et al., 1998		1:100	1:1000
TSHR	Mouse anti-human	Abcam	#ab6047	1:100	1:1000


### Image Acquisition and Analysis

**FIGURE 1:**
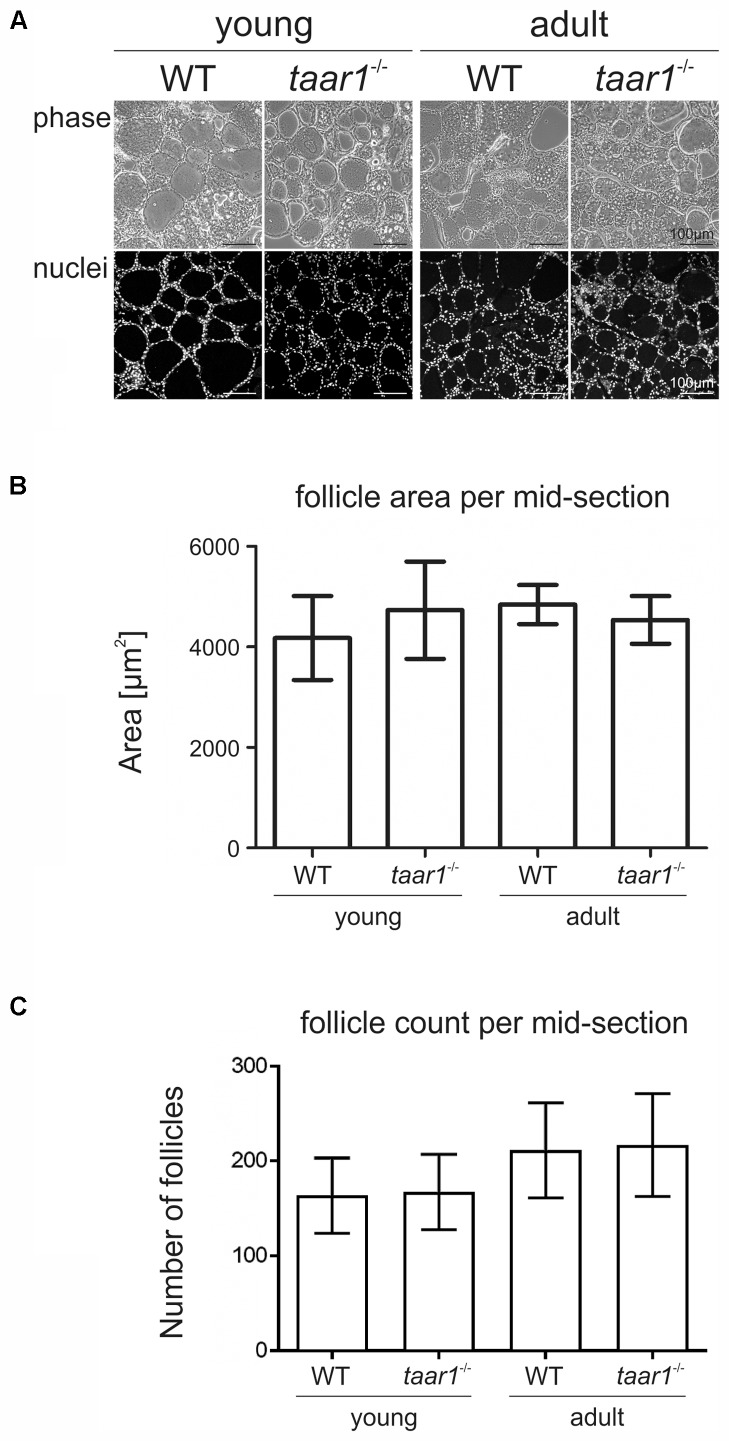
Morphometry of thyroid lobes revealing no gross alteration upon Taar1 deficiency. Cryosections through thyroid tissue obtained from young or older adult C57BL/6 WT and *taar1*^-/-^ mice **(A)** were analyzed by semi-automated morphometry through a Cell Profiler-based pipeline (Weber et al., 2015). Average follicle area per thyroid mid-section denotes the area covered by the thyroid follicles **(B)**. Follicle area is defined as the external edge of the follicular epithelia, i.e., the collagen IV-positive basal lamina encircling the thyroid follicle lumen. Follicle counts per thyroid mid-section are depicted in **(C)**. Note that there were no differences in follicle areas, but changes were observed in the number of follicles per thyroid mid-section in *taar1*^-/-^ and WT genotype [*F*(1,16) = 0.051, *p* = 0.824] but a genotype independent [*F*(1,16) = 0.002, *p* = 0.966] decrease in young vs. older adult mice [*F*(1,16) = 5.471, *p* = 0.033]. Phase contrast and corresponding single channel fluorescence micrographs of Draq5^TM^-counterstained nuclei of thyroid tissue are displayed in **A** as indicated. Scale bars represent 100 μm.

**FIGURE 2:**
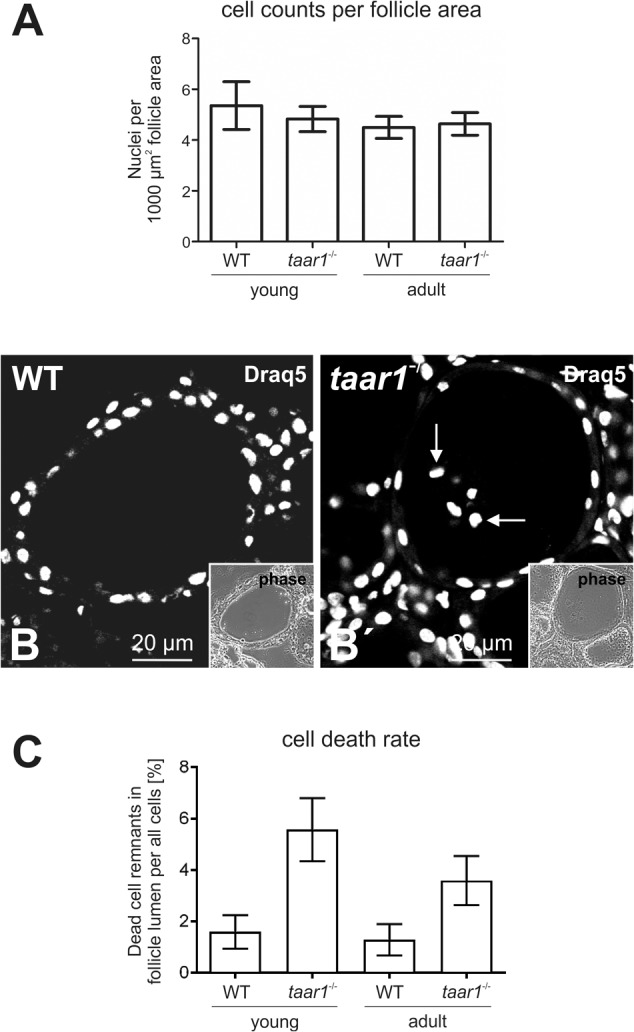
Number of cells per thyroid mid-section declines upon Taar1 deficiency, consistent with a higher cell death rate in Taar1-deficient follicles. Cryosections through thyroid tissue obtained from young or older adult C57BL/6 WT and *taar1*^-/-^ mice were analyzed by semi-automated morphometry through a Cell Profiler-based pipeline (Weber et al., 2015). The number of cells is given per 1,000 μm^2^ area of thyroid mid-section (average counts ± SD) in **(A)**. Note that young *taar1*^-/-^ mice were characterized by fewer numbers of cells per thyroid mid-section than corresponding WT controls, while there were no differences in the numbers of thyrocytes per 1,000 μm^2^ tissue area observed for older adult *taar1*^-/-^ and WT mice. Single channel fluorescence micrographs of Draq5^TM^-stained nuclei and corresponding phase contrast micrographs of thyroid follicular epithelia from young WT and *taar1*^-/-^ mice are depicted in **(B)** and **(B′)**, respectively. Note that remnants of dead cells were found to be present in follicle lumina of *taar1*^-/-^ but mostly absent from WT controls (**B′**, arrows). Scale bars represent 20 μm. The average percentage of dead cells (±SD) was found to be significantly higher in *taar1*^-/-^ thyroid epithelia than in WT controls [*F*(1,15) = 51.260, *p* < 0.001] **(C)**. There is a clear trend toward a decreased cell death rate in older mice [*F*(1,15) = 6.809, *p* = 0.020], independent of the genotype [interaction term *F*(1,15) = 3.636, *p* = 0.076].

**FIGURE 3:**
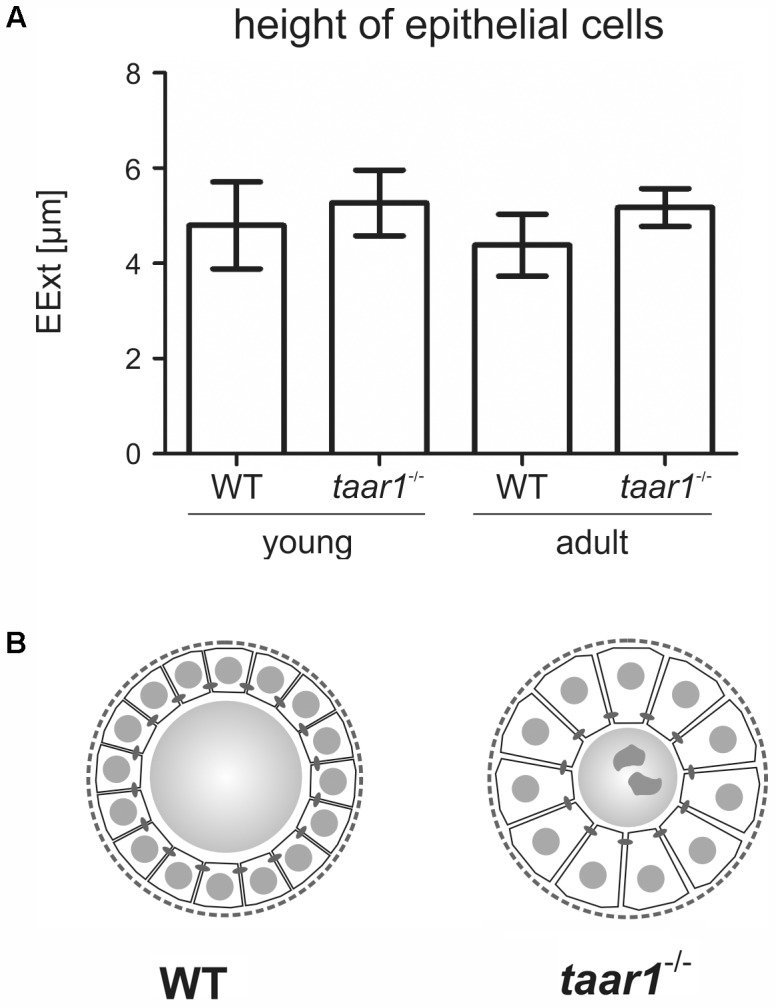
Thyroid follicular epithelia are more extended in mice with Taar1 deficiency. Cryosections through thyroid tissue obtained from young or older adult C57BL/6 WT and *taar1*^-/-^ mice were analyzed by semi-automated morphometry through a Cell Profiler-based pipeline (Weber et al., 2015). Epithelial extensions (EExts) were determined per thyroid mid-section **(A)**. Note that EExts tend to be higher in *taar1*^-/-^ mice when compared to the WT controls, thus implying *taar1*^-/-^ thyrocytes to be more prismatic, possibly indicating a higher activation state in the thyroid gland upon Taar1 deficiency. The scheme in **(B)** represents a comparison in sizes of thyrocytes relative to follicle lumen diameters in WT and *taar1*^-/-^ mice, respectively, not drawn to scale.

**FIGURE 4:**
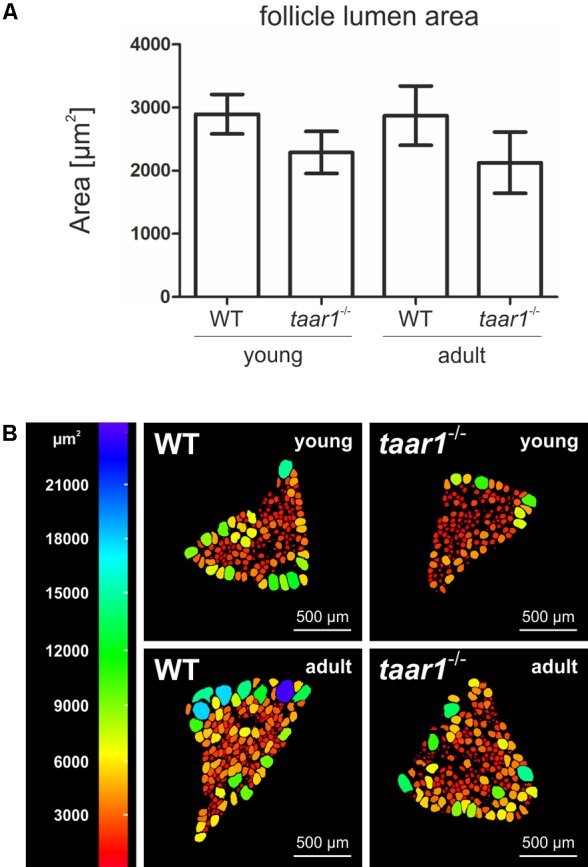
Follicle lumen areas decrease upon Taar1 deficiency. Cryosections through thyroid tissue obtained from young or older adult C57BL/6 WT and *taar1*^-/-^ mice were analyzed by semi-automated morphometry through a Cell Profiler-based pipeline (Weber et al., 2015) to determine the follicle lumen areas per thyroid mid-section. Note that the average luminal area, depicted as means ± SD, was smaller in *taar1*^-/-^ mice when compared to the WT controls **(A)** [*F*(1,16) = 14.421, *p* = 0.002]. A color-coded depiction of luminal area distribution per thyroid mid-section **(B)** shows considerable heterogeneity among the follicle population per thyroid section, with bigger follicles tending to localize on the thyroid lobe periphery, thus surrounding smaller, more centrally located follicles. This pattern of follicle distribution is maintained in both genotypes and similar for thyroid lobes from young and older adult mice, respectively. Scale bars represent 500 μm.

**FIGURE 5:**
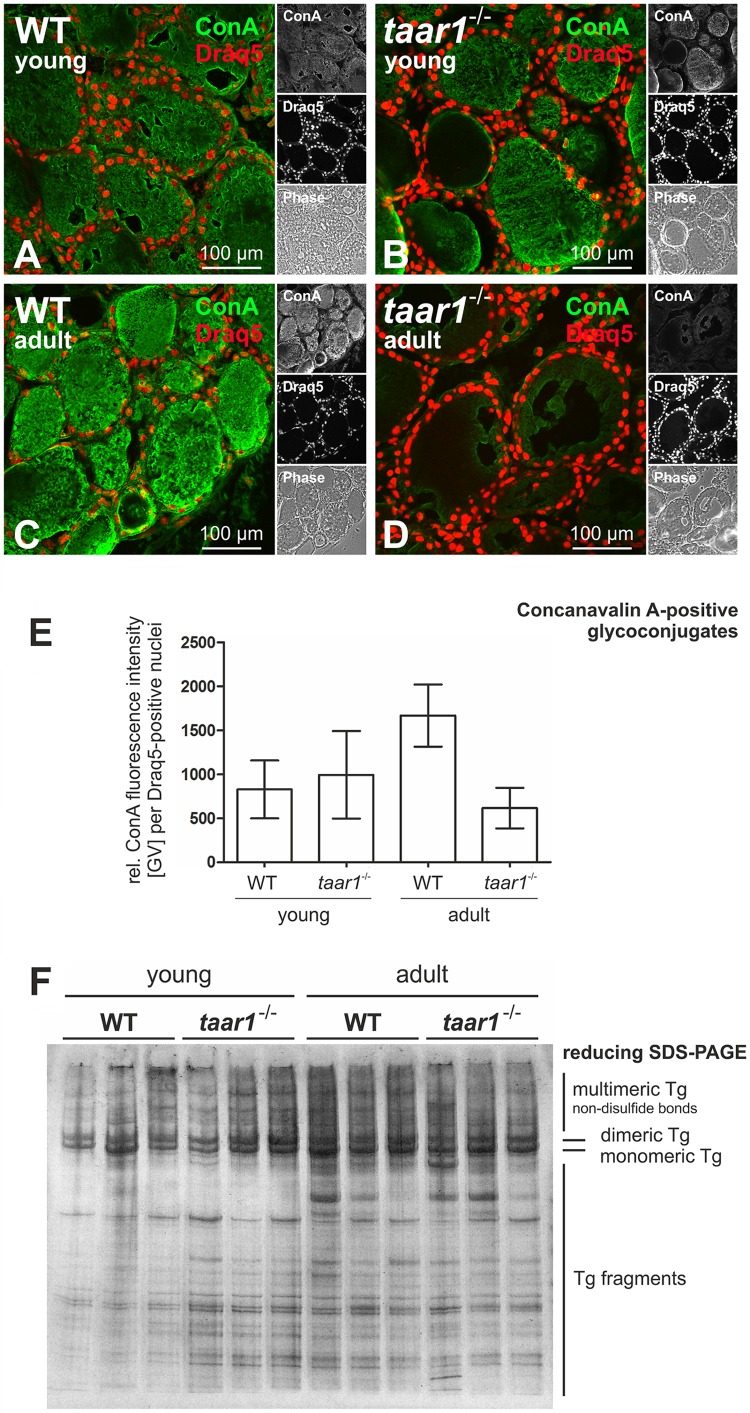
Protein glycosylation is reduced in older adult Taar1-deficient mouse thyroid tissue, while gross Tg degradation states are not affected. Cryosections through thyroid tissue obtained from young and older adult WT C57BL/6 and *taar1*^-/-^ mice were stained with the lectin ConA in order to determine the glycosylation status of luminal Tg (**A–D**, green). Nuclei were counter-stained with Draq5^TM^ (red signals). Merged, single channel fluorescence and corresponding phase contrast micrographs are depicted as indicated. Scale bars represent 100 μm. The fluorescence intensities of lectin staining of glycosylated tissue components of young and older adult mice of both genotypes, respectively, as indicated were determined through a Cell Profiler-based pipeline and normalized to the numbers of cells **(E)**; data are depicted in bar charts as means ± SD. Protein lysates prepared from young or older adult WT C57BL/6 and *taar1*^-/-^ mice, as indicated, were separated by SDS-PAGE under reducing conditions on a horizontal gel, which was silver-stained. The relative positions of bands representing multi-, di-, and monomeric Tg, as well as Tg fragments of lower molecular masses, are indicated in the right margin. Note that changes in glycosylation states were prevalent in thyroid tissue from young vs. older adult *taar1*^-/-^ mice and older adult WT vs. *taar1*^-/-^ mice (cf. **B** with **D**, and **C** with **D**, respectively, and **E**), but did not affect the extent or pattern of Tg degradation, which was comparable between *taar1*^-/-^ and WT mice **(F)**.

**FIGURE 6:**
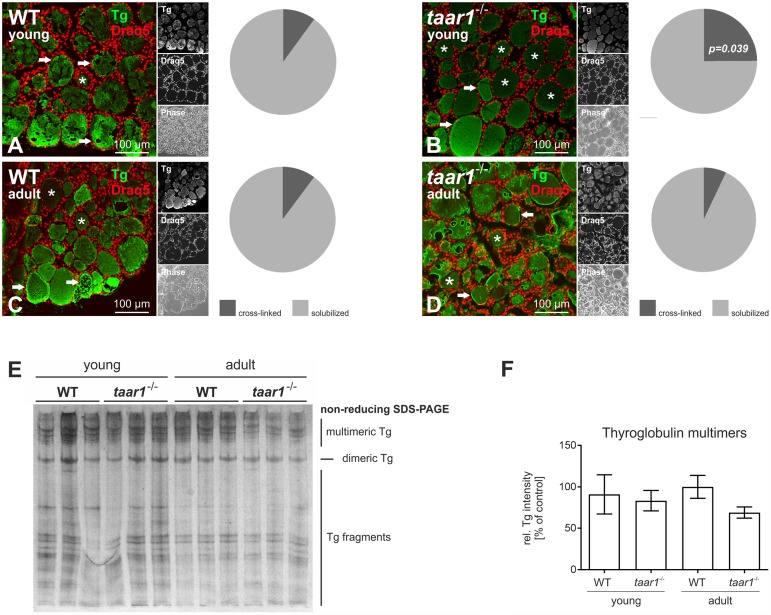
Thyroid follicles of *taar1^-/-^* mice present a difference in Tg storage capacity. Morphological assessment of intra-luminal Tg was performed by immunolabeling thyroid cryosections from young and older adult WT C57BL/6 and *taar1*^-/-^ mice with antibodies against Tg. The homogeneous, dimmer signal denotes cross-linked Tg (**A–D**, asterisks), as opposed to a higher-intensity labeling of Tg, owing to more accessible Tg epitopes for antibody binding, reflect the multilayered, partially solubilized Tg (**A–D**, arrows). At 24.9%, the prevalence of cross-linked Tg-containing follicle lumina was highest in the young *taar1*^-/-^ thyroid tissue, as compared to 10.0% in the young WT, and 10.2% and 7.0% in the older adult WT and *taar1*^-/-^, respectively **(A–D)**. Proteins isolated from young or older adult WT C57BL/6 and *taar1*^-/-^ mice were separated under non-reducing conditions by SDS-PAGE followed by silver staining **(E)**. Note that the less prominent signal of silver staining representing Tg multimers observed for older adult *taar1*^-/-^ **(E)**, also represented in the non-significant reduction of multimeric Tg band intensities **(F)**. Data are depicted as means ± SD.

**FIGURE 7:**
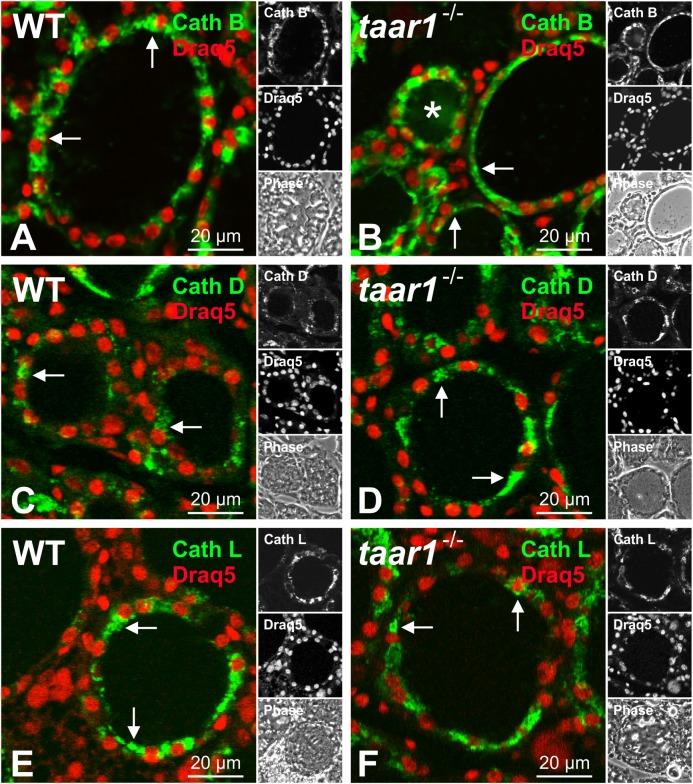
Subcellular localization of Tg-processing cathepsins remains unchanged in mouse thyroid gland epithelia upon Taar1 deficiency. Cryosections through thyroid tissue obtained from C57BL/6 WT and *taar1*^-/-^ mice were stained with antibodies against cathepsin B **(A,B)**, cathepsin D **(C,D)**, and cathepsin L **(E,F)**, and analyzed by confocal laser scanning microscopy. Single channel fluorescence micrographs in right panels: top cathepsin B, D, or L, as indicated, middle Draq5^TM^, bottom phase contrast. Images represent data obtained from young adult mice, only. Note that subcellular localization of cathepsins was not altered upon Taar1 deficiency and was mainly confined to endo-lysosomal compartments (**A–F**, arrows). Scale bars represent 20 μm.

Immuno- and lectin-labeled thyroid tissue cryosections were inspected with a confocal laser scanning microscope equipped with Argon and Helium-Neon lasers (LSM 510 Meta; Carl Zeiss Jena GmbH, Jena, Germany). Images were obtained at a pinhole setting of 1 Airy unit and at a resolution of 1024 × 1024 pixels. Per each biological replica, three to ten micrographs were chosen arbitrarily and analyzed with the LSM 510 software, release 3.2 (Carl Zeiss Jena GmbH). The number of biological replicas analyzed for ConA-staining (**Figures 5A–E**) were *n* = 3, 5, 3, and 3 for young WT, young *taar1*^-/-^, older adult WT, and older adult *taar1*^-/-^, respectively. The number of anti-Tg stained follicles morphometrically analyzed (**Figures 6A–D**) was *n* = 264 from four biological samples, *n* = 434 from six biological samples, *n* = 487 from three biological samples, and *n* = 346 from three biological samples for young WT, young *taar1*^-/-^, older adult WT, and older adult *taar1*^-/-^, respectively. The number of biological replicas used for analyses of cathepsin localization (**Figure 7**) were *n* = 3, 5, 4, and 3 for young WT, young *taar1*^-/-^, older adult WT, and older adult *taar1*^-/-^, respectively. The number of biological replicas used for analyses of cystatin C (**Figures 9B–D**), luminal cystatin D staining intensities (**Figures 9E–G**), anti-Mct8 and anti-Tshr localizations (**Figure 11**) were *n* = 3, respectively, for each genotype and age group. The epithelial extensions (EExts), follicle areas, follicle counts, follicle luminal areas, cell numbers per 1,000 μm^2^ of tissue area, as well as the fluorescence intensities of anti-cystatin C- and D-, anti-Tg, and ConA-positive signals were analyzed with the aid of the open source software Cell Profiler (version 2.1.1.; available from the Broad Institute at www.cellprofiler.org, Lamprecht et al., 2007), following our established pipelines described elsewhere (Weber et al., 2015). For determination of EExts (**Figure 3A**), analyses of follicle areas (**Figure 1B**), and determination of cell counts per follicle area (**Figure 2A**), the number of biological replicas analyzed was *n* = 4, 4, 4, and 3 for young WT, young *taar1*^-/-^, older adult WT, and older adult *taar1*^-/-^, respectively, with 208, 270, 275, and 200 total number of technical replicas, i.e., follicles, per experimental group, respectively. For enumerating follicles per mid-section (**Figure 1C**) and for determinations of follicle lumen areas (**Figure 4A**), the number of replicates was *n* = 7 from four biological replicas for the young WT, *n* = 6 from five biological replicas for the young *taar1*^-/-^, *n* = 4 from three biological replicas for the older adult WT, and *n* = 3 from three biological replicas for older adult *taar1*^-/-^, respectively. Number of replicas analyzed regarding cell death rates (**Figure 2C**) was *n* = 6, 7, 4, and 3 biological replicas for young WT, young *taar1*^-/-^, older adult WT, and older adult *taar1*^-/-^, respectively, while three to six different images were analyzed from each animal.

### Tissue Lysate Preparation

Resected deep-frozen thyroid tissue was homogenized on ice in lysis buffer (0.5% Triton X-100 in PBS, pH 7.4), and incubated at 4°C for 30 min. Lysates were cleared by centrifugation at 4°C and 13,000 × *g* for 5 min. Quantitative protein determination was performed by the Neuhoff method (Neuhoff et al., 1979) using BSA dissolved in 0.5% Triton X-100 in PBS as a standard.

### SDS-PAGE, Immunoblotting, and Silver Staining

Protein lysates were separated through SDS-PAGE on 12.5% self-cast vertical polyacrylamide gels along with a Page Ruler Prestained Protein ladder (Thermo Scientific, #26616), and transferred onto nitrocellulose membranes by semi-dry blotting. Unspecific binding sites were blocked by incubation with 5% blotting grade milk powder in PBS, supplemented with 0.3% Tween (PBS-T) for 16 h at 4°C. Afterwards, membranes were incubated for 2 h at room temperature with antibodies specific for cathepsins B, D, and L, as well as β-tubulin for normalization, each diluted in PBS-T, respectively (**Table 1**). Incubation with horseradish peroxidase-conjugated secondary antibodies (Southern Biotech, Birmingham, AL, United States, #6160-05, #4050-05, 1:5000) was performed for 1 h at room temperature, followed by visualization by chemiluminescence ECL Western Blotting substrate onto XPosure films (Pierce via Thermo Scientific, Schwerte, Germany). Band densitometry analysis of anti-cathepsin B, L, and D-positive bands was performed using ImageJ version 1.48, whereby the number of biological replicas was *n* = 3 for each genotype and age group, respectively.

A total of 0.5 μg protein from thyroid lysate preparations were separated on horizontal SDS Gradient 8-18 ExcelGel (GE Healthcare, Upsala, Sweden). Gels were silver stained (Heukeshoven and Dernick, 1988), and band densitometry analysis was performed on Image Studio Lite version 5.2 (LI-COR Biosciences GmbH, Bad Homburg, Germany) using *n* = 3 biological replicas for each genotype and age group, respectively.

### Cathepsin B Activity Assays

Cathepsin B activity assays were performed as described (Barrett, 1980; Brix et al., 1996; Mayer et al., 2009). In brief, protein samples prepared from thyroid tissues of *n* = 3 biological replicas per each genotype and age group, respectively, were assayed in triplicates by monitoring cleavage of 10 μM cathepsin B-specific substrate *N*-benzyloxycarbonyl-argininyl-arginine-7-amido-4-methylcoumarin hydrochloride (Z-Arg-Arg-AMC^∗^HCl; Bachem, Bubendorf, Switzerland, #I-1135) at pH 6.0, and for 60 min at 40°C. In negative controls, prepared for each sample, 10 μM E-64 were added at the start of the reaction time. Substrate cleavage was stopped by the addition of *2* M Tris-HCl (pH 9.0). The amounts of released AMC were quantified by measuring the fluorescence with a Tecan GENios Reader (Tecan Deutschland GmbH) using an excitation wavelength of 360 nm and emission reading at 465 nm.

### Measurement of Serum TSH Concentrations

Blood was taken from right heart ventricles, and allowed to clot by placing on ice before sera were stored at -20°C upon clearance by centrifugation at 4°C at 13,000 × *g* for 10 min. Blood serum samples were used for evaluation of TSH concentrations in duplicates or triplicates by use of a mouse TSH-specific ELISA kit (Cloud-Clone Corp. via Antibodies Online, # CEA463MU). Absorbance was determined at 450 nm using a Tecan Infinite M1000 Pro instrument (Grödig, Salzburg, Austria). For the serum TSH concentrations, *n* = 5 and 4 for young WT and young *taar1*^-/-^, respectively, and *n* = 3 and 5 for older adult WT and older adult *taar1*^-/-^, respectively, were used.

According to the manufacturer’s instructions, the detectable TSH concentrations range from 49.4 pg/ml to 4,000 pg/ml, with a sensitivity of 19.2 pg/ml. The manufacturer reports on the coefficients of variation (CV) as follows, namely CV < 10% for intra-assay and CV < 12% for inter-assay values, which were calculated as standard deviation (SD)/mean ^∗^ 100.

### Measurement of Total T_4_ and T_3_ Serum Concentrations

Total T_3_ and T_4_ serum concentrations were measured in duplicates by competitive radioimmunoassay (DRG Instruments GmbH, Marburg, Germany, #4525, #4524) according to the manufacturer’s instructions and as described (Wirth et al., 2015). Biological replicas were *n* = 7, 14, 11, and 6 for young WT, young *taar1*^-/-^, older adult WT, and older adult *taar1*^-/-^, respectively. The assay range, sensitivity and reference range with this mouse strain and narcotics were resp. 0.38–5.00 nM, 0.16 nM and 1.0–2.3 nM for T_3_, and 25–200 nM, 20 nM and 30–65 nM for T_4_. The intra-assay CV was ≤5% for both assays, and inter-assay CV 7.0% for T_3_ and 5.2% for T_4_.

### Statistical Analyses

Data are depicted as means ± SD. Because we have observed before that TH concentrations, i.e., free T_4_, in the blood serum of mice can decline upon older age (Friedrichs et al., 2003), we investigated the values as a function of age, and genotype. Statistical analyses were performed by conducting two-way ANOVAs using SPSS 24.0, while correlations are presented as Spearman’s rho. Data sets on protein quantification were compared for genotypic effects only using a Student’s *t*-test. Values of *p* < 0.05 were considered statistically significant.

## Results

### No Morphological Alterations Were Detectable Regarding Follicle Size and Follicle Count Between WT and *taar1^-/-^* Thyroids

To determine possible roles of Taar1 during development, which might be related to differentiation of thyroid follicle cells and thyroid tissue morphogenesis, a morphometric approach was chosen that allows determination of entire thyroid lobes and their constituency, namely the thyroid follicles (Weber et al., 2015). Initial morphological assessment revealed no significant differences in tissue morphology and follicle areas of either age, namely young and older adult mice or genotype, *taar1*^-/-^ mice and WT controls (**Figure 1**). The number of follicles per lobe in young animals was decreased (*p* = 0.033), independent of the genotype. Similarly, when comparing the average number of cells constituting a follicle, measured as the number of nuclei per 1,000 μm^2^ of follicle area, no significant difference was found, although the number of cells constituting thyroid follicles tends to be slightly higher in the young WT than in the other tested groups (**Figure 2A**). There were 4.81 ± 1.81 cells and 5.45 ± 2.24 cells per 1,000 μm^2^, which composed the follicles of young *taar1*^-/-^ and WT mice, respectively. Thus, the follicles of young *taar1*^-/-^ mice were rather comparable to follicles of older adult mice of both genotypes, which generally have less cells occupying 1,000 μm^2^ follicle area than per young WT follicle (**Figure 2A**).

### Lower Follicle to Cell Volume Ratios in *taar1^-/-^* Thyroids

The lack of Taar1 did not appear to have an effect on the tissue volume of either young or older adult mouse thyroids, as indicated by comparable follicle sizes and follicle counts. However, the finding of slightly fewer cells per follicle area in *taar1*^-/-^ vs. WT thyroid glands prompted us to further investigate and compare the rate of cell death and the volume of epithelial cells in *taar1*^-/-^ vs. WT. The mechanism of cell death in the thyroid gland is not fully understood, but it resembles terminal differentiation and shedding of dead cells into the lumen where the remnants remain detectable for long time intervals (Friedrichs et al., 2003; Nilsson and Fagman, 2017). In keeping with this notion, activation of procaspase 3 was not observed in WT or *taar1*^-/-^ thyroid tissue (data not shown). However, the proportion of dead cells was determined by counting the luminal Draq5^TM^-positive signal of nuclei representing such remnants of dead cells (**Figures 2B,B**′), divided by the total count of cells (nuclei) comprising the respective follicle. The results showed a significant rise in cell death rate in young *taar1*^-/-^ follicular epithelia, equivalent to 3.5-fold that of young WT, besides a 2.8-fold increase in the cell death rate of older adult *taar1*^-/-^ thyroid epithelia compared to the older adult WT, while cell death rates were comparable in both young and older adult WT follicular epithelia (**Figure 2C**).

Moreover, a trend toward higher EExts was revealed in both young and older adult *taar1*^-/-^ mice (5.27 μm ± 0.69 μm and 5.17 μm ± 0.40 μm, respectively) as compared to their WT counterparts (4.80 μm ± 0.91 μm and 4.38 μm ± 0.65 μm for young and adult WT, respectively; *F*(1,11) = 2.945, *p* = 0.114) (**Figure 3A**). This increase in the EExt is consistent with a decrease in the luminal area in *taar1*^-/-^ follicles, where average luminal area in young *taar1*^-/-^ thyroid follicles is significantly smaller (2,287 μm^2^ ± 334 μm^2^), when compared to their WT counterparts (2,895 μm^2^ ± 312 μm^2^) (**Figure 4A**). A similarly smaller luminal area was observed in the older adult *taar1*^-/-^.

The luminal areas were additionally investigated to provide a color-coded depiction of area distribution of follicular lumina per middle-section of a given thyroid lobe (**Figure 4B**). Such analyses highlighted the heterogeneity of the follicle populations per thyroid section, where the bigger follicles tend to localize on the thyroid lobe periphery, surrounding the smaller, more centrally located follicles. This distribution is maintained in both the young and older adult populations of thyroid follicles in both genotypes.

Overall, an approximate 10% and 18% increase in EExt in young and older adult *taar1*^-/-^ follicles, respectively, is indicative of a more prismatic thyrocyte shape (**Figure 3B**), suggesting a higher activation state (Friedrichs et al., 2003). Thus, higher thyrocyte activity states correlating to smaller thyroid follicle lumina could be associated with decreased Tg synthesis and/or increased secretion rates, or less colloid stored in the lumen, which, in turn, could be the outcome of enhanced Tg degradation and/or more compacted Tg. Therefore, we next performed biochemical and morphological analyses of Tg, while also conducting investigations on Tg-processing proteases and the availability of the TSH receptor in thyroid tissue of both genotypes.

### Taar1 Deficiency Has an Age-Dependent Effect on Thyroglobulin Glycosylation, But No Overall Effect on Its Synthesis Rate or State of Proteolytic Processing

Tg is a complex, heavily glycosylated molecule, which makes up most of the protein content of thyroid tissue. We therefore sought to visualize and compare the processing pattern of thyroidal Tg and to analyze its glycosylation pattern in both young and older adult WT and *taar1*^-/-^ thyroid tissue. The degree of Tg glycosylation was evaluated *via* quantification of fluorescence intensity of positive signal from thyroid cryosections stained with ConA (**Figures 5A–E**), a lectin that recognizes α-D-mannosyl α-D-glucosyl groups (Goldstein et al., 1974). The results indicated that Tg tends to be more glycosylated in older adult vs. young WT tissue, and less so in older adult *taar1*^-/-^ vs. WT glands (**Figure 5E**).

The degree of glycosylation is typically positively correlated with the degree of protein stability; therefore, we next checked the degradation status of Tg with the aim to answer whether or not Tg from older adult WT thyroids was less prone to degradation. A silver staining of SDS-PAGE separated proteins in thyroid lysates under reducing conditions shows no inter-genotypic differences in the amounts and gross degradation pattern of Tg (**Figure 5F**). However, overall degradation of thyroidal proteins appears to be enhanced in the older adult mice, as compared to their young counterparts.

On the other hand, morphological assessment of Tg storage was enabled by immunolabeling WT and *taar1*^-/-^ thyroid cryosections of both young and older adult mice with an antibody specific for Tg (see also Weber et al., 2017). The proportion of follicles with a homogeneous Tg-immunopositive signal, resembling intact cross-linked Tg, was higher in young *taar1*^-/-^ thyroid cryosections (10.0% and 24.9% for WT and *taar1*^-/-^, respectively), as opposed to the multi-layered, partially solubilized Tg, which makes it better accessible for antibody binding. This difference was no longer detected in the older adult mice, with the percentage of cross-linked Tg-containing follicles amounting to 10.2% and 7.0% in the WT and *taar1*^-/-^, respectively (**Figures 6A–D**).

Furthermore, protein separation under non-reducing conditions revealed a trend toward less Tg multimers in the older adult *taar1*^-/-^, as shown by the relative intensity of bands pertaining to multimeric Tg to the total protein per lane (**Figures 6E,F**, respectively). Because no difference in the amounts of Tg, a proxy for biosynthesis of Tg, was detected in thyroid lysates of WT and *taar1*^-/-^ thyroid tissue by reducing SDS-PAGE, a reduction in multimeric Tg forms in the lumen may reflect a difference in Tg storage capacity upon Taar1 deficiency.

Thus, the results show differences in Tg-glycosylation upon aging in both WT and *taar1*^-/-^ mice, as well as in Tg storage in cross-linked form in *taar1*^-/-^, in comparison to their WT counterparts. Hence, we next asked whether there was a difference in the efficiency with which Tg was solubilized, a process that is enabled by cathepsin B- and L-mediated extracellular proteolysis of covalently cross-linked Tg (Friedrichs et al., 2003).

### Altered Proteolytic Activity of Tg-Processing Cathepsins in the *taar1^-/-^* Thyroid

We first looked at whether or not Taar1 deficiency had any effect on the subcellular localization of cathepsins. Immunofluorescence revealed the subcellular localization of Tg-processing cathepsins B, D, and L to be unaltered, as they confine mainly to endo-lysosomal compartments in both WT and *taar1*^-/-^ thyroid tissue (**Figure 7**).

**FIGURE 8:**
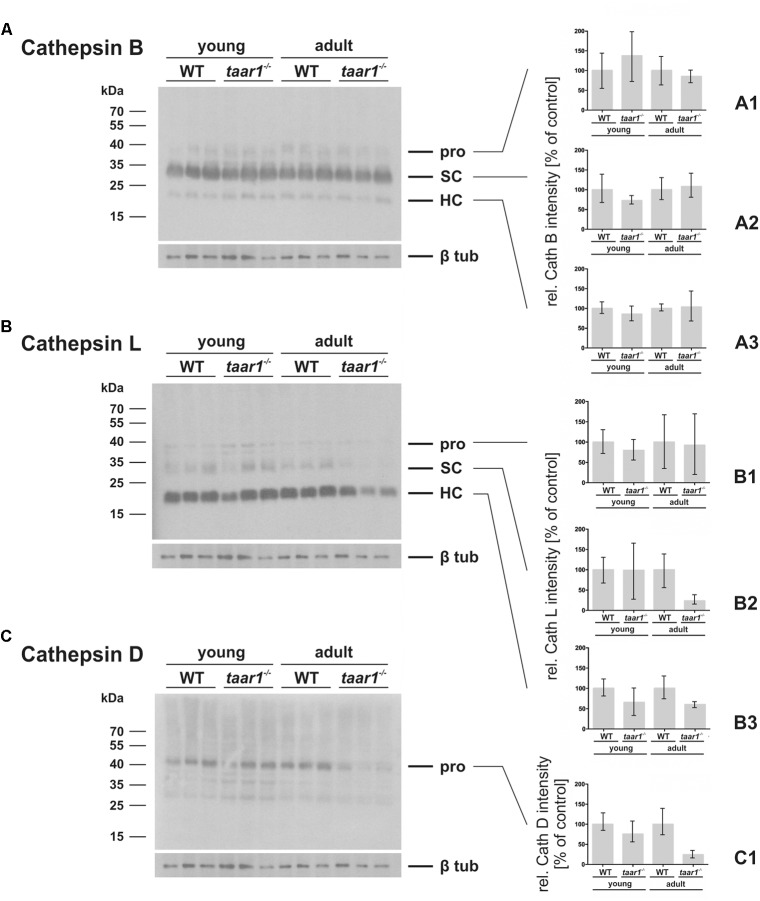
Variations in protein amounts of cathepsin B, L, and D in mouse thyroid gland upon Taar1 deficiency. Proteins were isolated from thyroid tissue obtained from young or older adult C57BL/6 WT and *taar1*^-/-^ mice, and separated by SDS-PAGE, followed by immunoblotting with antibodies against cathepsins B, L, or D. Protein amounts of the proform (pro), single chain (SC) and heavy chain of two-chain mature forms (HC) of cathepsin B **(A–A3)** and cathepsin L **(B–B3)**, and the proform of cathepsin D **(C,C1)** were analyzed by densitometry and normalized to β-tubulin. Molecular mass markers are indicated in the left margins **(A–C)**. Protein amounts of all forms of cathepsin B were not altered in *taar1*^-/-^ thyroids in comparison to WT controls. Two chain mature form protein amounts of cathepsin L were significantly decreased in *taar1*^-/-^ thyroids in comparison to WT controls [Genotype *F*(1,8) = 7.646, *p* = 0.024; age *F*(1,8) = 0.874, *p* = 0.377; interaction *F*(1,8) = 0.199, *p* = 0.667]. Similarly, the proform of cathepsin D was reduced in older adult *taar1*^-/-^ thyroids, as compared to WT controls **(C1)**, although not reaching the threshold of statistical significance. Data are depicted as means ± SD.

**FIGURE 9:**
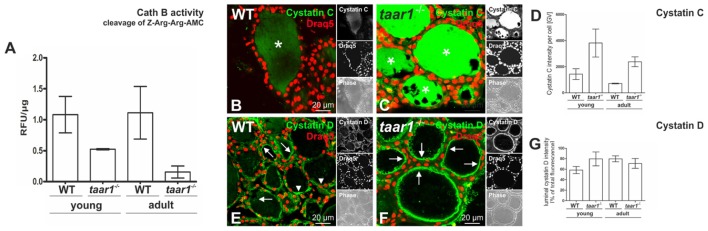
Proteolytic activity of cathepsin B is reduced in older adult mouse thyroid glands upon Taar1 deficiency, consistent with an increase in cystatin levels. Proteins were isolated from thyroid tissue obtained from young or older adult C57BL/6 WT and *taar1*^-/-^ mice, and proteolytic activity of cathepsin B was determined. The graph represents cleavage of Z-Arg-Arg-AMC^∗^HCl **(A)**. Cathepsin B activity levels of each sample were obtained by calculating relative fluorescence units (RFU) per protein concentration. Note that cathepsin B activity is genotype-dependently decreased in *taar1*^-/-^ mice in comparison to WT controls, irrespective of the age [Genotype *F*(1,8) = 24.944, *p* = 0.001; age *F*(1,8) = 1.256, *p* = 0.295; interaction *F*(1,8) = 1.727, *p* = 0.225]. Cryosections through thyroid tissue obtained from young or older adult C57BL/6 WT and *taar1*^-/-^ mice, were labeled with antibodies against cystatin C or D, and analyzed by confocal laser scanning microscopy **(B,C,E,F)**. Single channel fluorescence micrographs in right panels: top cystatin C or D as indicated, middle Draq5^TM^, and bottom phase contrast. Images represent data obtained from young mice. The graph in **(D)** represents quantification of the fluorescence intensity of cystatin C-positive signals per cell given in gray values (GV) for *taar1*^-/-^ mice vs. WT control [Genotype *F*(1,8) = 11.262, *p* = 0.010; age *F*(1,8) = 3.173, *p* = 0.113; interaction *F*(1,8) = 0.353, *p* = 0.569]. The graph in **(G)** represents quantification of fluorescence intensity of cystatin D-positive signals in the lumen, depicted as percentage of total fluorescence intensity of cystatin D per cell for *taar1*^-/-^ mice vs. WT control for both ages [Genotype *F*(1,8) = 1.368, *p* = 0.276; age *F*(1,8) = 1.412, *p* = 0.269; interaction *F*(1,8) = 7.989, *p* = 0.022]. Cystatin C was mainly localized in the follicle lumen (**B,C**, asterisks) in both WT and *taar1*^-/-^ mice. Note that protein amounts of cystatin C were increased in *taar1*^-/-^ mice in comparison to WT controls. Cystatin D was mainly localized to the peri-cellular space of the lumen **(E,F)** in both WT and *taar1*^-/-^ mice (arrows), while arrowheads point toward intracellular cystatin D-positive vesicles. Scale bars represent 20 μm. Data are depicted as means ± SD. Levels of statistical significance are indicated as ^∗^ for *P* < 0.025.

Furthermore, densitometric analysis of immunoblots showed both the pro- and mature forms of cathepsin B to be comparable in WT and *taar1*^-/-^ thyroid tissue (**Figures 8A–A3**). However, a slight decrease in the single chain cathepsin L was noted in adult *taar1*^-/-^ thyroids, while retaining comparable levels of both the pro- and heavy chain of cathepsin L in both age groups of both genotypes (**Figures 8B–B3**). Similarly, a slight reduction in cathepsin D proform was observed in adult *taar1*^-/-^ thyroid lysates (**Figures 8C,C1**). Despite no differences in total cathepsin B protein levels, however, an age-effect was elucidated in the proteolytic activity of this protease, namely that it was significantly diminished in older adult *taar1*^-/-^ thyroid lysates compared to their young counterparts (**Figure 9A**), that latter also exhibiting lower proteolytic activity than in the WT controls.

The notion of reduced Tg-solubilizing capacity in Taar1 deficiency is further supported by an enhancement in the fluorescence intensity of cystatin C immunopositive signal in *taar1*^-/-^ thyroid cryosections (**Figures 9B–D**), cystatin C being an endogenous inhibitor of cysteine proteases. Moreover, the related cystatin D was also localized intra-luminally in the peri-cellular space at the apical plasma membrane, where extracellular proteolysis for Tg solubilization and initial T_4_ liberation occurs, with an approximate 36% increase in the fluorescence intensity of luminal cystatin D observed in the young, but not in older adult *taar1*^-/-^ thyroid cryosections, as compared to the WT (**Figures 9E–G**).

Overall, subtle differences in the balance of proteolytic to anti-proteolytic activities are observed in the *taar1*^-/-^ mouse thyroid gland with declining potency to solubilize and degrade Tg by both, extra- and intra-cellular means. In order to see whether this had a systemic effect, we next looked at T_3_, T_4_, and TSH concentrations in the serum.

### Lack of Functional Taar1 Leads to a Mild Case of Hyperthyrotropinemia in Young Adult Mice

**FIGURE 10:**
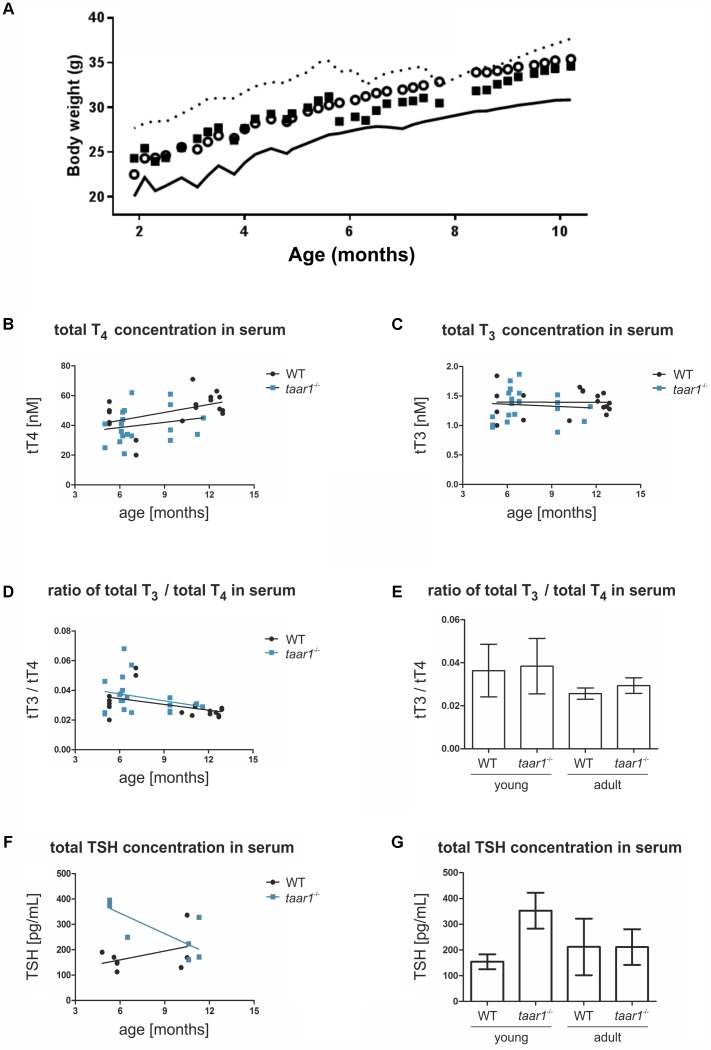
Elevated serum TSH concentrations in young taar1^-/-^ mice, while body weight gain and thyroid hormones concentrations remain comparable to the WT. Body weight gain was quantified between 8 and 44 weeks of age, revealing no difference between WT and *taar1*^-/-^ mice **(A)**. Data are depicted as circles representing means – SD plotted as continuous line below for the WT, and as squares representing means + SD plotted as dotted line above for *taar1^-/-^*. Total T_4_ **(B)** and total T_3_ **(C)** serum concentrations of *taar1*^-/-^ vs. WT mice were quantified by radioimmunoassay. Data showed total T_4_ and T_3_ serum concentrations were not altered in *taar1*^-/-^ mice in comparison to WT controls (**B,C**; WT *r* = 0.420, *p* = 0.083; Taar1 *r* = 0.370, *p* = 0.109; and WT *r* = -0.086, *p* = 0.726; Taar1 *r* = 0.023, *p* = 0.916, respectively). Ratios of total T_3_ over total T_4_ serum concentrations were not altered in *taar1*^-/-^ vs. WT mice but declined at older age in both genotypes **(D,E)** [WT *r* = -0.445, *p* = 0.064; Taar1 *r* = -0.169, *p* = 0.477; and Genotype *F*(1,34) = 0.751, *p* = 0.392; age *F*(1,34) = 8.735, *p* = 0.006; interaction *F*(1,34) = 0.060, *p* = 0.809]. Serum TSH was determined by ELISA, revealing an age-dependent effect of the genotype on TSH concentration in the serum [WT *r* = -0.037, *p* = 0.931; Taar1 *r* = -0.062, *p* = 0.908; and Genotype *F*(1,34) = 8.417, *p* = 0.012; age *F*(1,34) = 1.529, *p* = 0.238; interaction *F*(1,34) = 8.554, *p* = 0.012]. The TSH of young *taar1*^-/-^ mice is increased, as compared to the age-matched WT controls **(F,G)**. Data are depicted as scatter plots including trendlines in **B–D** and **F**, and the bar graphs in **E** and **G** display means ± SD.

To analyze whether the observed disbalances in Tg proteolysis in the thyroid gland of Taar1-deficient mice had a systemic effect, the animals were weighed over a time period of 8–44 weeks of age, revealing no change in body weight gain between WT and *taar1*^-/-^ mice (**Figure 10A**).

Thyroid hormone serum concentrations were measured by radioimmunoassay, revealing an age-dependent increase in total T_4_ concentrations in both genotypes, with a trend toward lower total T_4_ in young *taar1*^-/-^ mice. Age-related increasing T_4_ and stable T_3_ serum concentrations were comparable in both genotypes (**Figures 10B,C**).

The ratios of T_3_ over T_4_ were determined to deduce potential differences in TH metabolism and release from thyroid follicles. Thus, we analyzed whether the T_3_ over T_4_-ratios were changed in blood serum, in order to detect symptoms of stimulated deiodinase activity in thyroid tissue of young adult Taar1-deficient animals, which were potentially hypothyroid. However, the T_3_ over T_4_-ratios were not altered in the blood serum of *taar1*^-/-^ vs. WT thyroid tissue in animals of both age groups (**Figures 10D,E**), indicating that preferential T_3_ release from the thyroid follicles of *taar1*^-/-^ mice due to altered intra-thyroidal TH metabolism did not occur. However, decreased T_3_ over T_4_-ratios in older adult mice in comparison to their young counterparts revealed decreased T_4_ to T_3_ conversion rates at older age (*p* = 0.006) (**Figures 10D,E**).

To complete the picture, serum TSH concentrations were measured *via* ELISA revealing an increase in WT and a decline of serum TSH concentrations in *taar1*^-/-^ mice with older age (**Figure 10F**). The increase in serum TSH concentrations was significant when comparing young adult *taar1*^-/-^ mice with their WT counterparts (**Figure 10G**).

**FIGURE 11:**
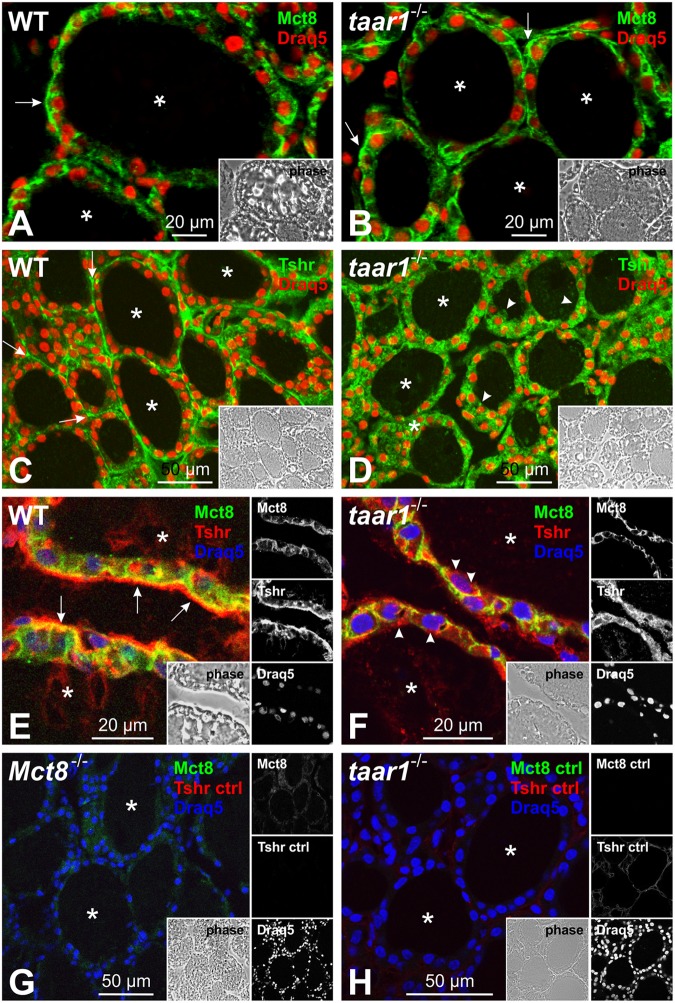
Taar1 deficiency alters Tshr localization in the mouse thyroid glands, but does not affect Mct8 localization. Cryosections through thyroid tissue obtained from young C57BL/6 WT and *taar1*^-/-^ mice were labeled with antibodies against TSHR and Mct8 and examined by confocal laser scanning microscopy. Mct8 maintains a basolateral localization in both WT and in *taar1*^-/-^ thyroid tissue (**A,B**, arrows), while Tshr is more prominently localized in intracellular vesicles of *taar1*^-/-^ thyrocytes in young mice (**D,F**, arrowheads), as opposed to predominantly basolateral distribution in the WT (**C,E**, arrows). **(E,F)** present a close-up on thyroid epithelia of WT and in *taar1*^-/-^ mice, respectively, as merged Tshr and Mct8 images. Secondary antibody controls are shown in **(G,H)** as indicated. Single channel fluorescence micrographs in right panels: top Mct8, middle Tshr, bottom Draq5^TM^ as nuclear counter-stain. Insets represent corresponding phase contrast micrographs. Images represent data obtained from 5 to 7 months old mice. Scale bars represent 20 μm in **(A,B,E,F)** and 50 μm in **(C,D,G,H)**.

Furthermore, the basolateral localization of Mct8, the major TH-exporting molecule of thyrocytes, was not altered in *taar1*^-/-^ vs. WT thyroid tissue (**Figures 11A,B**), thus also ruling out major differences in T_4_ release from thyroid follicles as a cause of high TSH concentrations in the serum of young Taar1-deficient mice.

Finally, we checked whether the TSH receptor (Tshr) localization was affected in the *taar1*^-/-^ thyroid tissue. Immunofluorescence inspection of thyroid sections from young mice labeled for Tshr showed a stark difference in the receptor’s localization between the genotypes, where it was predominantly localized to vesicular compartments in *taar1*^-/-^ tissue, as opposed to the classical basolateral pattern of subcellular localization in the WT tissue (**Figures 11C–F**).

Taken together with the observation of largely unaffected TH metabolism, the data point to a mild state of altered feedback regulation in young *taar1*^-/-^ mice. We conclude that Taar1 deficiency does not lead to primary hypothyroidism, despite the observed changes in the proteolytic network. Rather, delocalized Tshr signify a thus far non-identified resistance syndrome, in particular in the young *taar1*^-/-^ mice, which are characterized by hyperthyrotropinemia.

## Discussion

Members of the TAAR family of receptors such as TAAR1 have been proposed as targets mediating the actions of the TH metabolite 3-T_1_AM (Panas et al., 2010). Albeit several actions of 3-T_1_AM are distinct from those of classical TH if administered in pharmacological doses, only few studies addressed the role of TAAR1/Taar1 and its ligand 3-T_1_AM on the thyroid gland and the regulation of the HPT axis (Klieverik et al., 2009; Agretti et al., 2011; Schanze et al., 2017). Recently, Schanze et al. (2017) have shown that the *in vivo* application of 3-T_1_AM led to the decrease in expression of the sodium-iodide symporter (*Nis*), *pendrin* and *Tg*, but did not alter *tshr* expression, nor did it affect TSH-induced signaling in the treated animals, leading the authors to conclude that stimulation with 3-T_1_AM does not affect the HPT axis but interferes with functional characteristics of thyroid epithelial cells. The results of that study may suggest that the 3-T_1_AM-triggered effects are indeed not Taar1-mediated (Schanze et al., 2017). One could also argue, however, that this may be simply because Taar1 is not present at the basolateral plasma membrane domain of thyrocytes, and cannot be activated by circulating 3-T_1_AM. However, it is not known yet whether in thyrocytes Taar1 gets activated by either thyronamines or other TH metabolites such as 3-T_1_AM or 3-T_1_Ac. Indeed, 3-T_1_AM can be metabolized by thyrocytes to yield T_0_AM and 3-T_1_Ac, as shown by Schanze et al. (2017) for PCCL3 cells, or might reach the thyroid follicular lumen by means of transcytotic transport as previously suggested (Szumska et al., 2015). While the latter proposal has not been tested experimentally, the present study supports the view that Taar1 is indeed involved in thyroid gland morphogenesis and in maintenance of regular thyroid states. Similar to the observations in *taar1*^-/-^ mice of this study, a smaller follicular lumen and higher epithelial heights have been observed after administration of 3-T_1_AM for 7 days in male mice (Schanze, 2017). The results of the present study and those upon 3-T_1_AM administration therefore suggest a role of both Taar1 and circulating 3-T_1_AM in maintaining thyroid follicle architecture and functionality of thyrocytes.

Here, we show that the thyroid gland of *taar1*^-/-^ mice is characterized by more prismatic epithelial cells and, consequently, smaller follicle lumen areas in which the cross-linked, compact form of Tg is stored (**Table 2**). Such a phenotype is typically observed in TSH-activated thyroid glands, where peripheral TH demands are feeding back on the TH-generating cells to first, liberate TH for short-term supply of target organs, and second, trigger re-synthesis of Tg to fill up the stores of TH precursor molecules. A question that has been asked for a long time, refers to the mechanisms by which individual follicles are able to sense the amount of stored Tg, because an autonomy in thyroid follicle activity is observed for any given thyroid follicle in conditions of TH demand or proper TH supply alike (Roger et al., 1992; reviewed in Suzuki et al., 2011). An involvement in such regulatory processes by primary cilia at the apical plasma membrane domain of thyrocytes, and reaching out into the Tg stored within the thyroid follicle lumen, has been discussed before but was formally never proven (Herzog et al., 1992; reviewed in Brix et al., 2001).

**Table 2:** Summary of phenotypic changes quantified in *taar1^-/-^* vs. WT male mice of different age groups.

	Phenotypic changes of *taar1*^-/-^ male mice vs. WT	
	
	5–8 months	10–15 months
Follicle area per mid-section	=	=
Follicle count per mid-section	=	=
Cell counts per follicle area	↓	=
Cell death rate	↑↑↑	↑
Height of epithelial cells	↑	↑
Follicle lumen area	↓↓	↓
ConA-positive glycoconjugates	=	↓↓
Tg degradation pattern (reducing SDS-PAGE)	=	=
Tg cross-linkage (immunostaining)	↑	=
Tg multimerization (non-reducing SDS-PAGE)	=	**↓**
protein amounts of Tg-processing cathepsins	Cath B =	Cath B =
	Cath L =	Cath L ↓
	proCath D =	proCath D **↓**
Proteolytic activity of Tg-processing cathepsins	Z-Arg-Arg-AMC cleavage ↓	Z-Arg-Arg-AMC cleavage ↓↓
Protein amounts of cystatin C per cell	↑↑	↑
Protein amounts of luminal cystatin D	↑	=
Total T_4_ serum concentration	=	=
Total T_3_ serum concentration	=	=
Total T_3_ over T_4_-ratio in serum	=	=
Total TSH serum concentration	↑↑	=


### Taar1 Is Important for Thyroid Morphology and Function

Our results (**Table 2**) show that the absence of functional Taar1 imposes no overt changes in the thyroid gland morphogenesis, nor does it affect the overall size of the gland. However, closer inspection revealed increased cell death rates in *taar1*^-/-^ thyrocytes, which, although statistically proven insignificant, show a trend toward more extended thyroid epithelia, resulting in significantly smaller follicular lumen area. Such changes were found to be more pronounced in the younger group of tested animals. Taar1 deficiency also presents subtle disturbances in the levels and proteolytic activity of Tg-processing cathepsins, resulting in persistence of more cross-linked Tg in the follicle lumen of young animals. Moreover, a correlation can be drawn between the increase in cell death rate in the *taar1*^-/-^ and the decrease in the amounts of mature cathepsin L, since cathepsin L is regarded as a survival factor for thyrocytes, whereupon cathepsin L-deficient thyroid follicles were marked by highly prominent cell remnants in the follicular lumen (Friedrichs et al., 2003).

Decreased cathepsin B activity and smaller amounts of cathepsin L were observed in *taar1*^-/-^ follicles (**Table 2**). Judging by the fact that cathepsin B activity and the amount of cathepsin L were only mildly diminished in the *taar1*^-/-^ thyroids vs. WT, but higher cystatin levels prevailed, we conclude that Taar1 deficiency might have limited effects on the expression of thyrocyte-typical genes, but might rather affect proteostasis, resulting in altered balances of proteolytic to anti-proteolytic activities in *taar1*^-/-^ vs. WT controls. Hence, the mildly misbalanced Tg turnover observed upon Taar1 deficiency is likely due to alterations in the extents of extra- vs. intracellular cathepsin-mediated Tg processing. Because cathepsin trafficking in thyrocytes is regulated by TSH stimulation (Linke et al., 2002), future studies will have to entail a better understanding of intrathyroidal auto-regulation and how this is connected to TSH-regulation *via* the HPT axis and/or to intrathyroidal Taar1 signaling in individual thyroid follicles.

### Canonical Regulation of the Thyroid Gland Upon Taar1 Deficiency

Although thyroid-specific ligands remain unknown, it is clear that the Taar1 receptor has a role in thyroid gland regulation involving the HPT axis, because in the absence of functional Taar1, the localization and potentially the signaling outcome of ligand-triggered Tshr are affected. The obvious abundance of the Tshr in intracellular compartments of *taar1*^-/-^ thyrocytes, as indicated by the predominantly vesicular Tshr-immunopositive signals, and as opposed to a mainly basolateral localization of Tshr in the WT, could be due to either poor targeting of the Tshr to the cell surface, which would lead to partial TSH resistance (Calebiro et al., 2005), or to an enhancement in Tshr internalization. The latter is the inevitable fate of an activated GPCR to be internalized into endo-lysosomal compartments, generally a β-arrestin-chaperoned and clathrin-mediated pathway, from where the receptor is either recycled back to the cell surface, or proceeds to be degraded by endo-lysosomal enzymes (reviewed in Luttrell and Lefkowitz, 2002). Receptor internalization has initially been regarded as a process exclusive for GPCR down-regulation (reviewed in Lohse, 1993); however, it is also essential for efficient receptor desensitization (reviewed in Calebiro, 2011).

In the case of the TSHR, it is co-internalized complexed to its ligand into subcellular compartments, even including retrograde trafficking to the *trans*-Golgi network (Godbole et al., 2017), from where it continues to persistently signal *via* the cAMP-related pathways (Frenzel et al., 2006; Calebiro et al., 2009, 2010; Werthmann et al., 2012; Godbole et al., 2017). It is noteworthy that downstream cellular targets of cAMP signaling appear to be dependent on the subcellular site of signal origin (Rich et al., 2001; Godbole et al., 2017), i.e., GPCRs signaling from within intracellular compartments do not necessarily activate the same pathways as classically activated by cell surface-localized signaling GPCRs.

One must also consider the possibility of Tshr undertaking an alternative trafficking route, leading to less Tshr reaching the surface in the first place, resulting in a lesser proportion of Tshr being available for classical TSH stimulation. Either scenario may ultimately result in less efficient TH liberation *via* cathepsin-mediated Tg-proteolysis. Future studies will have to clarify the molecular mechanistic pathways explaining such interconnections of cathepsin-mediated Tg-proteolysis, Taar1 and Tshr localization in individual thyroid follicles of rodent thyroid gland tissue. Testing such scenarios experimentally will become better accessible when using cellular models in which TSHR-bearing human thyroid epithelial cell lines express TAAR1 at the cell surface (Qatato et al., 2014).

## Conclusion and Perspectives

**FIGURE 12:**
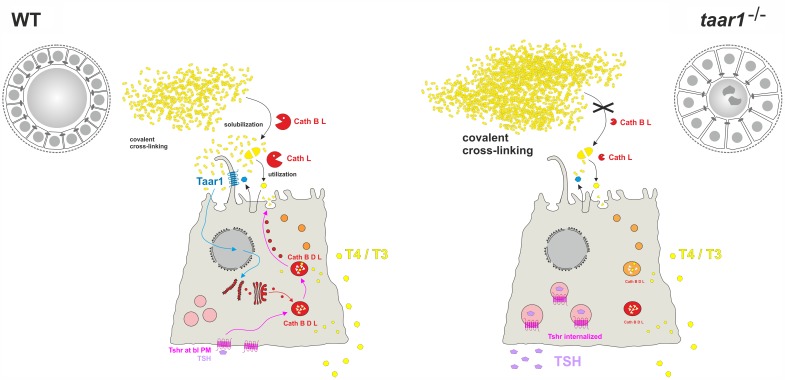
The influence of Taar1 deficiency on liberation of TH in thyroid follicles. Tg (yellow) is synthesized and secreted at the apical plasma membrane of thyrocytes for storage in covalently cross-linked form in the thyroid follicle lumen of WT (left). TSH (violet) binding to basolateral Tshr (pink) triggers retrograde trafficking of endo-lysosomal enzymes (red) for secretion at the apical pole into the peri-cellular follicle luminal space. Subsequently, Tg is solubilized extracellularly by the action of cathepsins B and L before being internalized for endo-lysosomal degradation (orange to red). TH release (yellow) from thyroid follicles is through balanced proteolytic processing of Tg by extra- and intra-cellular means of proteolysis, and subsequent Mct8-mediated translocation across the basolateral plasma membrane. This study asks whether Tg solubilization and processing to yield TH is possibly co-regulated by Taar1 (cyan), which is localized to cilia at the apical plasma membrane of thyrocytes where it can, in principle, interact with intra-follicular generated TH derivatives triggering signaling of this GPCR. Smaller thyroid follicle lumina in male *taar1*^-/-^ vs. WT mice reveal thyroglobulin storage in more compacted form (right). Enhanced luminal cystatins C and D (not depicted) render thyroglobulin-solubilizing cathepsin B (red) less active in thyroid tissue of *taar1*^-/-^ mice. Cathepsin L amounts (red) are diminished in *taar1*^-/-^ vs. WT thyroid follicles featuring more dead cell remnants (dark gray irregular shaped symbols) in the follicle lumen. More extended epithelia do not affect gross thyroid hormone (yellow polygons) release from thyroid follicles of *taar1*^-/-^ mice. TSH (violet) concentrations in the blood serum are enhanced upon Taar1 deficiency, while TSH receptors (pink) are non-canonically located in intracellular vesicles. The results indicate Taar1 is necessary to maintain canonical HPT-axis regulation of thyroid function in male mice.

Our results (**Figure 12** and **Table 2**) show that Taar1-deficient thyroid follicles are characterized by fewer cells per follicle area, owing to more prismatic epithelia and higher cell death rates, in addition to a decreased luminal area, in which the compacted cross-linked form of Tg is more prevalent than in the WT controls. These alterations are more evident in young (5–8 months) than in the older adult (10–15 months) animals.

We assumed that the increased cell death rates in *taar1*^-/-^ thyroid tissue correlate to diminished amounts of cathepsin L (see above), but might also hint toward self-thyrotoxicity due to altered TH metabolism caused by changes in deiodinase activities. Indeed, similar to the results reported in this study, decreased concentrations of serum T_4_, accompanied by normal or reduced serum T_3_ and increased serum TSH concentrations have been described for deiodinase 3 (D3) knock-out mice (for a recent review, see: van der Spek et al., 2017). However, different from *taar1*^-/-^ mice investigated in this study, young *D3*^-/-^ animals are typically hyperthyroid and become hypothyroid at older age. This is different from our observation with *taar1*^-/-^ mice, which exhibited no signs of primary hypo- or hyperthyroidism. Dropping T_3_ over T_4_-ratios were detected in blood serum of older vs. young adult *taar1*^-/-^ mice, possibly hinting to enhanced T_4_ generation and export at older age, which is however also typical for the WT animals and therefore not dependent on Taar1.

We conclude that Taar1-deficient animals show a mild TSH receptor resistance syndrome, which is due to the apparent loss of Tshr from the basolateral plasma membrane, most likely resulting in altered functionality. Most strikingly, the thyroid epithelia of young Taar1-deficient mice are characterized by a vesicular localization of the Tshr, which signifies that Taar1 signaling is necessary to maintain homeostatic Tshr signaling and canonical HPT-axis regulation (**Figure 12**).

In future, it will be important to investigate *taar1*^-/-^ mice with regard to possible delocalization of TSH receptors in extrathyroidal tissues. These are in particular adipose, bone and muscle tissues, all derived from the mesenchymal stem cell lineage, that are bearing functional TSHR/Tshr, and are thus directly affected by TSH-induced signaling in a TH-independent manner (for review, see: de Lloyd et al., 2010). Interestingly, also in TSHR/Tshr-expressing mesenchymal stem cells, TSH-triggered signaling seems to be different from the canonical cAMP pathway that is prevalent for the cell surface-localized TSHR/Tshr in the thyroid (de Lloyd et al., 2010; Godbole et al., 2017).

Overall, the data of this study are therefore in line with previous characterizations of *taar1*^-/-^ mice that did not exhibit a growth phenotype, or severe alterations in organogenesis, especially not with regard to classical TH target organs like the central nervous system or the liver (Wolinsky et al., 2007; Lindemann et al., 2008). The investigations herein highlight, however, that it will be critically important to better understand the process of Tg degradation and possible intrathyroidal thyronamine action (for discussion, see Szumska et al., 2015; Hoefig et al., 2016), to deduce thyroid follicle auto-regulatory mechanisms triggered by GPCRs like Taar1 at the cilia of thyrocytes *in situ*. In order to test this hypothesis, it would be required to analyze the composition of the peri-cellular luminal content with regard to TH derivatives, in particular, thyronamine precursors, and thyronamines themselves. Such investigations might become feasible in future with more advanced imaging MALDI spectrometry at hand.

Furthermore, the results of this study highlight the importance of evaluating Taar1-targeting drugs in pre-clinical studies for potential side effects they may have on thyroid gland homeostasis. Such pre-clinical studies would optimally consider newly emerging paradigms for diagnosis and treatment of thyroid disorders (Chatzitomaris et al., 2017; Hoermann et al., 2017), revealing more comprehensive tools to study set-point changes and adjusting pathways beyond classical HPT-axis regulation of thyroid function and thyroid states in individual patients.

## Author Contributions

MQ, JS, VS, ER, and KB performed the experiments. JK and KB devised the study and supervised the experimental work. All authors contributed to data interpretation and manuscript drafting. All authors read and approved the final manuscript.

## Conflict of Interest Statement

The authors declare that the research was conducted in the absence of any commercial or financial relationships that could be construed as a potential conflict of interest.
